# Plant acclimation to environmental stress: a critical appraisal

**DOI:** 10.3389/fpls.2015.00445

**Published:** 2015-06-12

**Authors:** Naser A. Anjum

**Affiliations:** CESAM-Centre for Environmental and Marine Studies, Department of Chemistry, University of AveiroAveiro, Portugal

**Keywords:** crop health, crop productivity, plant genetic engineering, sustainable agriculture, plant stress acclimation

Being a prime source of food, agriculture sustains almost all life-forms on the Earth. However, apart from natural calamities, a range of man-made activities are causing significant increase in environmental stresses for plant growth. Adverse conditions, in turn, restrict crop plants to reach their full genetic potential of producing high yield (Anjum et al., 2014). Also, agriculture is under pressure to accelerate crop-yield to feed rapidly increasing world-population that is projected to stabilize at around 9.2 billion toward the year 2050 (Singh, 2012). Thus, a clear understanding of the strategies for sustainably improving crop-health, development and productivity under adverse conditions is timely and imperative. With the major aim of achieving plant-environmental stress-acclimation, discussions are focused in the 17 chapters of the entitled above edited book mainly on two major tools: (a) genetically engineering stress-responsive genes, and (b) sustainable agriculture practices.

Under the categorized above first tool, the strategy of employing DREB-like proteins, homeobox genes, APETALA2 gene-family, and G-proteins for overcoming environmental-stresses and increasing crop-yield under adverse conditions. Notably, exhaustive studies on the potential role of the dehydration-responsive element-binding proteins (DREB2)-like proteins and their interaction with abscisic acid particularly during seed-germination/seedling-growth, may give us an insight in plant developmental processes and stress-adaptation/tolerance-mechanisms. A gradual rise in atmospheric temperature is going to be a major challenge for crop research in near future. To this end, exhaustive molecular genetic studies were recommended for both understanding and getting more insights into thermo-sensing-mechanisms in thermal-stressed crops. Nevertheless, development of several functional tools like molecular maps, express sequence tags (ESTs), and understanding the mechanism of transgenes expression in chloroplasts particularly in important bioenergy crops can help in the minimization of the rapidly increasing marginal lands, the contributions. Transcription factors (TFs) represent master-switches controlling several target stress-responsive genes and are considered most important for regulation of gene expression. Stress-tolerance in plants can be engineered through getting insights into homeobox-TFs and APETALA2/ethylene response element-binding protein (AP2/EREBP) in abiotic/biotic stress responses and their potential use as target genes. However, a detailed functional analysis of homeobox and AP2 TFs is required to identify novel pathways and better understand underlying molecular mechanisms.

Regulation of stress related pathways, including reactive oxygen species-production, stomatal regulation and processes related to plant water relations can be modulated by G-proteins and related machinery. G-proteins were suggested herein as the key proteins for overcoming environmental stresses and increasing crop yield. Elucidation of different non-genetic or epigenetic determinants that could modify gene expression heritably (mitotically and/or meiotically) and reversibly (without changing the gene sequence encoded in DNA) can enlighten different terminal phenotypes in genetically identical cells or even whole-organisms. Nevertheless, enzyme-mediated chemical-modifications of DNA and DNA-associated proteins are among the major epigenetic determinants/marks and can allow for greater genome-plasticity which in turn can result into better adaptation of plants to changing environmental-conditions. Additionally, oxidative stress-response and the pleiotropic roles of nitric oxide (NO) in signaling, gene-expression, and plant stress-tolerance can be accelerated with the genomic-dissection of available NOS-deficient mutant and/or gene knock-out mutant. Interestingly, compared to crop plants, weeds exhibit their higher tolerance to abiotic/biotic stresses, and can be an alternate and potential source of genes for the production of transgenic plants. Agricultural-soil-salinity is increasing globally, and is now known to contribute to major percentage of crop-loss. Cloning of genes from the halobacterium and most of its relatives were advocated to be significant for developing drought- and salinity-tolerant plants. Previous aspects were discussed in detail in the present edited volume.

A range of sustainable agriculture practices can be good assets to the crop plant genetic engineering research and help in achieving the common target of improving plant-acclimation to environmental stresses. In this context, contributions also advocated to use sustainable-strategies such as maintenance of rich agrobiodiversity, planting of varieties resistant to major insects, use of botanical-pesticides, bio-fertilizers (including mycorrhizae; vermicomposting), exploiting plant-pathogen interaction-outcomes, and intercropping and planting of different crops at any given time to spread the risk. Poor seed-germination and seedling-emergence has been the major cause of crop failure worldwide. Hence, it would be wise to use priming-compounds (such as NO, H_2_O_2_, H_2_S, polyamine), and beneficial microorganisms to achieve both improved germination/seedling-emergence, and induced stress-acclimation in these crops. Apart from food-crops, there is ample scope to improve active-constituents of several medicinal and aromatic plants growing under adverse conditions through exploiting key-mechanisms underlying Ca-regulated transcriptional genes and their role in stress-signaling pathways. Finally, consideration of a comparative account of the outcomes of controlled laboratory- and field-conditions was emphasized in the extensive evaluation of stress-tolerance potential of transgenic-crops with desirable traits.

To the negative side, much of the contributions simply summarized the literature and there is little attempt to provide an overarching critical assessment of the cited literature as a whole, or to draw new or insightful-conclusions. Also, inclusion of a final chapter on “Conclusions and perspectives” could have provided a novel and insightful synthesis of the literature in the field together with the promised critical-assessments of the recent research on both the previous tools considered separately herein.

Notably, in the present edited volume, the current knowledge for plant acclimation to environmental stresses was practically updated and the information on the major concerned areas was presented. Together, the discussion-outcomes can provoke new dialogue, and open scientific minds for innovative future research for the improvement of crop-health and productivity under adverse conditions. The book “*Plant acclimation to environmental stress*” may be a good information source for researchers, and PhD/MPhil students associated with crop-biology/physiology, agronomy and plant breeding areas.

This book can be found at: http://link.springer.com/book/10.1007%2F978-1-4614-5001-6

## Conflict of interest statement

The author declares that the research was conducted in the absence of any commercial or financial relationships that could be construed as a potential conflict of interest.
